# Copy Number Variation at the APOL1 Locus

**DOI:** 10.1371/journal.pone.0125410

**Published:** 2015-05-01

**Authors:** Rupam Ruchi, Giulio Genovese, Jessica Lee, Victoria T. Charoonratana, Andrea J. Bernhardy, Seth L. Alper, Jeffrey B. Kopp, Ravi Thadhani, David J. Friedman, Martin R. Pollak

**Affiliations:** 1 Division of Nephrology, Department of Medicine, Beth Israel Deaconess Medical Center, Boston, Massachusetts, United States of America; 2 Division of Nephrology, Department of Medicine, University of Florida, Gainesville, Florida, United States of America; 3 Harvard Medical School, Boston, Massachusetts, United States of America; 4 Stanley Center, Broad Institute of Harvard and MIT, Cambridge, Massachusetts, United States of America; 5 Broad Institute of Harvard and MIT, Cambridge, Massachusetts, United States of America; 6 Kidney Diseases Branch, National Institutes of Health, Bethesda, Maryland, United States of America; 7 Renal Unit, Department of Medicine, Massachusetts General Hospital, Boston, Massachusetts, United States of America; 8 Center for Vascular Biology Research, Beth Israel Deaconess Medical Center, Boston, Massachusetts, United States of America; Biogen Idec, UNITED STATES

## Abstract

Two coding variants in the APOL1 gene (G1 and G2) explain most of the high rate of kidney disease in African Americans. APOL1-associated kidney disease risk inheritance follows an autosomal recessive pattern: The relative risk of kidney disease associated with inheritance of two high-risk variants is 7–30 fold, depending on the specific kidney phenotype. We wished to determine if the variability in phenotype might in part reflect structural differences in APOL1 gene. We analyzed sequence coverage from 1000 Genomes Project Phase 3 samples as well as exome sequencing data from African American kidney disease cases for copy number variation. 8 samples sequenced in the 1000 Genomes Project showed increased coverage over a ~100kb region that includes APOL2, APOL1 and part of MYH9, suggesting the presence of APOL1 copy number greater than 2. We reasoned that such duplications should be enriched in apparent G1 heterozygotes with kidney disease. Using a PCR-based assay, we observed the presence of this duplication in additional samples from apparent G0G1 or G0G2 individuals. The frequency of this APOL1 duplication was compared among cases (n = 123) and controls (n = 255) with apparent G0G1 heterozygosity. The presence of APOL1 duplication was observed in 4.06% of cases and 0.78% controls, preliminary evidence that this APOL1 duplication may alter susceptibility to kidney disease (p = 0.03). Taqman-based copy number assays confirmed the presence of 3 APOL1 copies in individuals positive for this specific duplication by PCR assay, but also identified a small number of individuals with additional APOL1 copies of presumably different structure. These observations motivate further studies to better assess the contribution of APOL1 copy number on kidney disease risk and on APOL1 function. Investigators and clinicians genotyping APOL1 should also consider whether the particular genotyping platform used is subject to technical errors when more than two copies of APOL1 are present.

## Introduction

Individuals of recent African ancestry have a markedly increased risk of kidney disease and kidney failure compared with other groups. The high rate of kidney disease in African Americans has been shown to be largely due to two coding variants in the APOL1 gene[1, 2]. The two risk alleles include G1, comprising two non-synonymous coding variants rs73885319 (p.Ser342Gly) and rs60910145 (p.Ile384Met), and a 6 bp deletion (rs71785313) termed G2 (p.388-389delNY), both leading to changes in the protein sequence near the C-terminus. The association between these two APOL1 alleles and kidney disease has been widely replicated in a variety of samples sets and study designs (reviewed in [3]). Risk of kidney disease conferred by APOL1 G1 and G2 variants follows an essentially recessive mode of inheritance. The presence of one risk allele, as determined by standard genotyping methods, also seems to confer a much smaller but significant increase in the risk of disease (odds ratio 1.26 to 1.9)[1, 4, 5], and is also associated with a younger age of initiating dialysis[6, 7].

It is now in fact well recognized that copy-number changes, small insertions or deletions in the genome, are an extremely common form of variation that contributes significantly to phenotypic differences[8]. Upon review of data from the 1000 Genomes Project, we noted evidence of such structural variation at the APOL1 locus. We were interested in understanding this variation in greater details, as the existence of altered APOL1 structure in a subset of individuals may have implications for understanding genotyping results and alter the function and/or expression of APOL1. Characterizing this structural variant in APOL1 is the initial step towards understanding the possible contribution of structural variation to altered gene expression and/or function.

The biological behavior of the protein(s) generated by a single APOL1 may differ from an allele with a fully or partially duplicated APOL1 as well as a truncated or deleted APOL1. We therefore performed a preliminary investigation to see if copy number variation at the APOL1 might contribute to the phenotypic variability associated with specific APOL1 genotypes.

## Materials and Methods

### Sporadic cases and controls

Participants gave informed consent for these genetic studies. Research was conducted under a protocol approved by the institutional review board (the Committee on Clinical Investigations) at Beth Israel Deaconess Medical Center, Boston. All samples in this study were from African American individuals.

Cases and controls samples were selected from samples previously genotyped for APOL1 kidney disease risk alleles G1 and G2. For this study, we used only samples (cases) from kidney disease individuals with genotyping indicating apparent heterozygosity for G1 (i.e. genotype G0/G1). Of 123 cases, 25 had biopsy proven FSGS, the remainder had non-diabetic ESRD. The 255 controls were African American individuals with apparent genotype G0/G1 without known kidney disease. Genomic DNA from samples NA 19700, NA 19701, NA 19702, NA 19372 was extracted from the lymphocyte cell lines obtained from the Coriell Institute for Medical Research. The first 3 of these were a parent-child trio of African ancestry from the Southwest United States. Sample NA 19372 was from an individual of Luhya tribe from East Africa.

### DNA genotyping

In all samples, DNA quality was assessed with 260/280 optical density ratios. All samples were genotyped with respect to G0 wild type, G1 (rs73885319) and G2 (rs71785313) SNPs using custom designed TaqMan Allelic discrimination Assays. For most of the samples, genotyping by Sanger sequencing was also performed and was in agreement with Taqman results.

### Qualitative PCR assay for detection of duplication

Forward and reverse primers were designed to amplify a 291 bp region across the junction of the duplication, if present. A touchdown PCR with variable annealing temperatures was used to decrease the possibility of non-specific binding of primers to DNA templates. The PCR product was visualized by electrophoresis on a 3% agarose gel. A band of approximately 300 bp indicated the presence of the APOL1 duplication in that sample.

### Taqman copy number assay

The assay was performed using Applied Biosystems 7300 real time PCR system. Each PCR reaction consisted of 1 μl 20X custom copy number assay, 1 μl RNAse P assay (reference assay, run simultaneously), 10 μl Taqman Master Mix, 4 μl molecular grade water and 4 μl of genomic DNA (5 ng/μl). The copy number assay was custom designed to amplify and detect a 142 bp region in the last exon of APOL1, and consisted of a forward primer (5’-TTACCAACTCACACGAGGCATT 3”), reverse primer (5’-CTCCACCTGTTCACCGCTTT 3’) and FAM labeled probe (5’-ACATCCGTGCCCTCA3’). The RNase P assay was obtained from Life Technologies, and also consisted of a forward and reverse primer, as well as a VIC labeled probe, designed to amplify and detect RNase P H1 gene, known to exist in 2 copies in human genome. Each sample was run in quadruplicate. Each plate contained of three controls; one a sample with presumed 2 copies of APOL1 gene, the second a non-template control. The third control was determined to have 3 copies based on initial testing by copy number assay. The results were analyzed using Copycaller software version 2. Copycaller software v2.0 (Applied Biosystems). Only those samples where the copy number call was made with at least 80% confidence were included for final analysis (107 cases and 249 controls).

### Allelic Discrimination Digest Assay

PCR was performed using primers designed to amplify a 314 bp, G0/G1 containing region of APOL1 for allelic discrimination via restriction digest. 10 μL of each PCR product was digested with HindIII or NspI (cleaving G0 at S342G and G1 at I384M, respectively) according to manufacturer’s directions (New England Biolabs) for 3–4 hrs at 37C. The entire digest reaction volume was run on a 2% agarose gel. Gel images and band intensities were quantified with Flourchem Q image analysis system.

### Cloning PCR Fragments for Sequencing and Haplotype Identification

We amplified a region of the APOL1 gene containing the G1 and G2-defining variants (forward primers: 5’-GCCAATCTCAGCTGAAAGCG, reverse primer 5’-TGCCAGGCATATCTCTCCTGG). Undigested PCR products cloned into pCR 4-TOPO entry vectors and transformed into chemically competent TOP10 *E*. *coli* according to manufacturer’s protocols (Invitrogen K4575-01SC). Plasmid DNA was isolated and sequenced to identify clones with different G0/G1/G2 haplotypes.

### Statistical Analysis

Cases and controls were compared with respect to the result of their PCR assay as well as copy number (2 or 3) by Taqman copy number assay. P value was calculated by two-tailed fisher exact test, using Graphpad software.

## Results

### Data from 1000 Genomes Project

**Fig 1:**
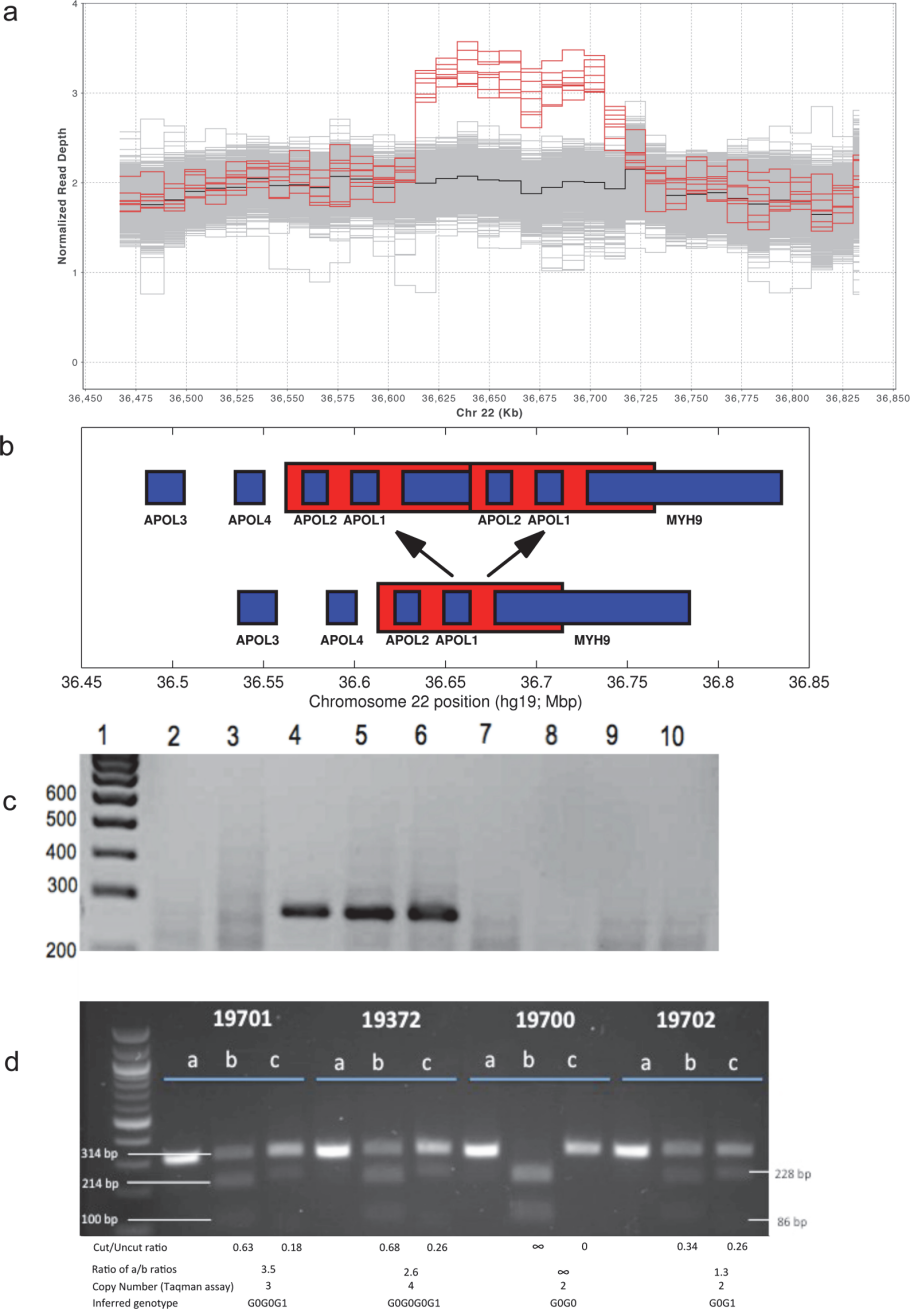
Copy number variation at APOL1 locus. a. Sequence coverage analysis of samples from 1000 Genomes Project. Grey lines are used to depict coverage of each of 2,527 samples at genomic loci to which sequence reads map. Red lines indicate coverage for 8 samples with apparent duplication. The black line depicts median coverage for all samples showing elevated coverage over a region of this locus, including APOL1, suggesting duplication. Coordinates of the genome are plotted on the X-axis against normalized read depth on the Y-axis. Coordinates of APOL1 gene are chr22: 36,649,117–36,663,571 (hg19). b. Pictorial representation of the duplication event. An approximately 100kb region of chromosome 22, beginning from a region adjacent to APOL2, and involving all of APOL1 and part of MYH9 is duplicated (chr22:36,612,938–36,714,262, hg19). c. Gel electrophoresis (3% agarose) of the PCR product of the qualitative PCR assay used for detection of duplication. The PCR assay amplifies sequence across the junction between the duplicated segments, and results in a 291 bp size product, if present. Lane 1 corresponds to 100 bp ladder with size of each fragment indicated. Lanes 2 and 3 represent samples from individuals of European ancestry, who are negative for duplication. Lanes 4, 5 and 6 show a band of the expected size and indicate the presence of a duplication in these three unrelated African American individuals. Lanes 7, 8 and 9 show the absence of a PCR product in three other African American individuals. One of these three (lane 7) showed 3 copies using the Taqman copy number assay, indicating an insertional variant of different structure. Lane 10 is a non-template control reaction. d. Allelic discrimination digest assay: Gel electrophoresis of the PCR products obtained during allelic discrimination assay. Lane 1 shows a 100 bp ladder. Lane 2 (a, b and c) corresponds to sample NA 19701, Lane 3 (a, b and c) corresponds to sample NA 19372, Lane 4 (a, b and c) corresponds to sample NA 19700 and lane 5 corresponds to sample NA 19702. The band in the lanes 2a, 3a, 4a and 5a represent PCR products not subjected to any digestion (314 bp in size). Lanes 2b, 3b, 4b and 5b represent PCR products after digestion with HindIII. Lanes 2c, 3c, 4c and 5c represent PCR products after digestion with NspI. Since sample NA 19700 is homozygous for G0, there are only 2 bands in lane 4b and 1 band in lane 4c. Of note, with HindIII digestion, which cleaves the PCR product only if G0 allele is present, bands are expected to be 100 bp and 214 bp in size. With NspI, which cleaves the PCR product only in the presence of I384M G1 allele, the bands are expected to be 228 and 86 bp in size.

Review of publicly available data in the 1000 Genomes Project Phase 3 dataset[9]vrevealed the presence of eight individuals (out of 2,527 total), all of African descent, with elevated sequence coverage over an approximately 100 kb region containing the APOL1 gene (Fig 1A). Compared to an average normalized read depth of 2, the read depth in this interval was approximately 3 in these individuals, suggesting the presence of heterozygosity for a small duplicated region that included the APOL1. Four of these 1000 Genomes samples have sequence evidence for the presence of the G1 allele (SNPs rs73885319 and rs60910145), one sample has evidence for the G2 allele (rs71785313), and three samples have evidence of neither.

Sequence reads spanning the breakpoints suggest that the most parsimonious explanation for this increase in coverage is due to a 101,324 bp long tandem duplication of the sequence block chr22:36,612,938–36,714,262 (GRCh37/hg19 coordinates), as illustrated in Fig 1B (see also S1 Table). The duplication does not seem to be mediated by non-allelic homologous recombination, although there is 4 bp micro-homology at the breakpoints (TCTG at chr22:36,612,938–36,612,942 and chr22:36,714,262–36,714,266), suggesting Microhomology-Mediated Break-Induced Replication (MMBIR) as the putative mechanism [10]. Haplotypes carrying this duplication would be expected to contain two copies of APOL1 and two copies of APOL2.

The telomeric breakpoint falls within exon 11 of the MYH9 gene, making it possible to detect reads spanning the breakpoint from exome sequencing data. Review of sequences from 13 African American exomes previously sequenced by our laboratory identified two related individuals with similar or identical duplication events (S1 Table). We reviewed previous Affymetrix SNP 6.0 genotyping data from these two individuals. Analysis of this data for copy number variation using PennCNV[11] provided additional confirmation of the presence of this duplication. Similarly, previous large-scale efforts to catalog CNVs throughout the genome suggested the presence of a duplication at this locus[12].

We sequenced the ~291bp PCR amplified DNA fragment used to assay the presence of this duplication using as template genomic DNA from ten individuals with this APOL1 duplication. The DNA sequence of this fragment was identical in these ten individuals, indicating identical breakpoints. Due to the presence of the identical APOL1 locus breakpoint in ten different unrelated individuals of recent African ancestry, its absence from any individuals of European descent in the 1000 Genomes Project, and the relatively frequent presence of this duplication in individuals with the G1 allele that originated in Africa, we believe the presence of this duplication is the result of a single event that took place in Africa. Since APOL1 alleles G1 and G2 have reached high frequency in West Africa as a result of natural selection it is interesting to speculate whether the duplication event might have a beneficial effect[13]. Since the current model predicts an increased risk for kidney disease with G1 and G2 in the absence of G0, a haplotype carrying two copies of APOL1 would reduce the chances of G0 being completely absent in a G1 or G2 positive individual.

### Genotyping

We designed a PCR based assay constructed to amplify the breakpoint region. In the presence of this duplication, the primers will amplify a 291 bp region across the junction between the two copies. Samples identified by whole genome/exome sequencing as positive and negative for duplication were used as controls for this assay Fig 1C).

Based on 1000 Genomes data, this APOL1 duplication is present on 8 of 1382 African APOL1 alleles (allele frequency ~0.57%), and 0 of 3688 non-African alleles. Because there has been small but fairly consistently observed increased rate of kidney disease in individuals genotyped as heterozygous for the G1 or G2 risk alleles (but much smaller than the risk for those with two copies of G1 or G2), we were interested in the possibility that a non-trivial subset of individuals with apparent G0G1 genotype may have a third copy of APOL1 due to this chromosomal rearrangement that could increase risk of disease.

To determine if presence of this duplication correlated with disease in heterozygous individuals (i.e. if apparent G0G1 heterozygotes by standard genotyping had additional copies of APOL1 at increased frequency), we used the PCR assay described above to genotype 123 African American patients with kidney disease (Table 1), and 255 African Americans without kidney disease, for the presence of this duplication. We found that 5 out of 123 cases (4.09%) were positive for the presence of an APOL1 duplication, as compared to 2 out of 255 controls (0.78%; p = 0.03 by Fisher’s exact test).

**Table 1:** Fraction of case and control samples in which an extra copy of APOL1 was detected by either a PCR assay designed to amplify across the insertional junction, or by a quantitative Taqman-based copy number assay.

*Assay*	*G0G1 cases (kidney disease)*	*G0G1 controls (no kidney disease)*	
PCR assay	5/123	2/255	p = 0.03
Taqman assay	CN2 94	CN2 241	p = 0.002
	CN3+ 11	CN3+ 6	
	No call 16	No call 6	

Next, we used a quantitative PCR assay (Taqman) to measure APOL1 copy number in these same samples, as an independent assay that might detect additional, different structural changes that would not be detected by the first PCR assay. We found that all of the samples that were positive for the APOL1 duplication by qualitative PCR had at least 3 APOL1 copies by Taqman copy number assay. However, we additionally found 6 cases and 4 controls that had 3 copies by Taqman assay but were negative for the duplication by the qualitative PCR assay. This suggests the presence of additional rare duplications in APOL1 that are structurally different from the first duplication identified. There were 22 samples in which calls could not be made with at least 80% confidence.

### Relationship of APOL1 duplication to kidney disease risk alleles

In theory, a tandem duplication of APOL1 could have several possible G0 vs G1 vs G2 genotypes. Specifically, the tandem APOL1 haplotype could be G0G0, G0G1 (or G1G0), or G1G1. As noted above, 8 samples from the 1000 Genomes Project Phase 3 were found to have 3 copies of APOL1 based on sequence data. Three of these individuals had only the G0 genotype, indicating that at least some of the duplicated alleles contained a tandem duplication of the G0 allele (genotype G0 / G0G0). In four of these samples, genotyping showed the presence of both the G0 and G1 alleles. Sequence reads that covered the G1 defining SNPs were too sparse to allow us to determine with certainty whether a given sample had 2 copies of G0 and 1 copy of G1, or 1 copy of G0 and 2 copies of G1.

**Fig 2:**
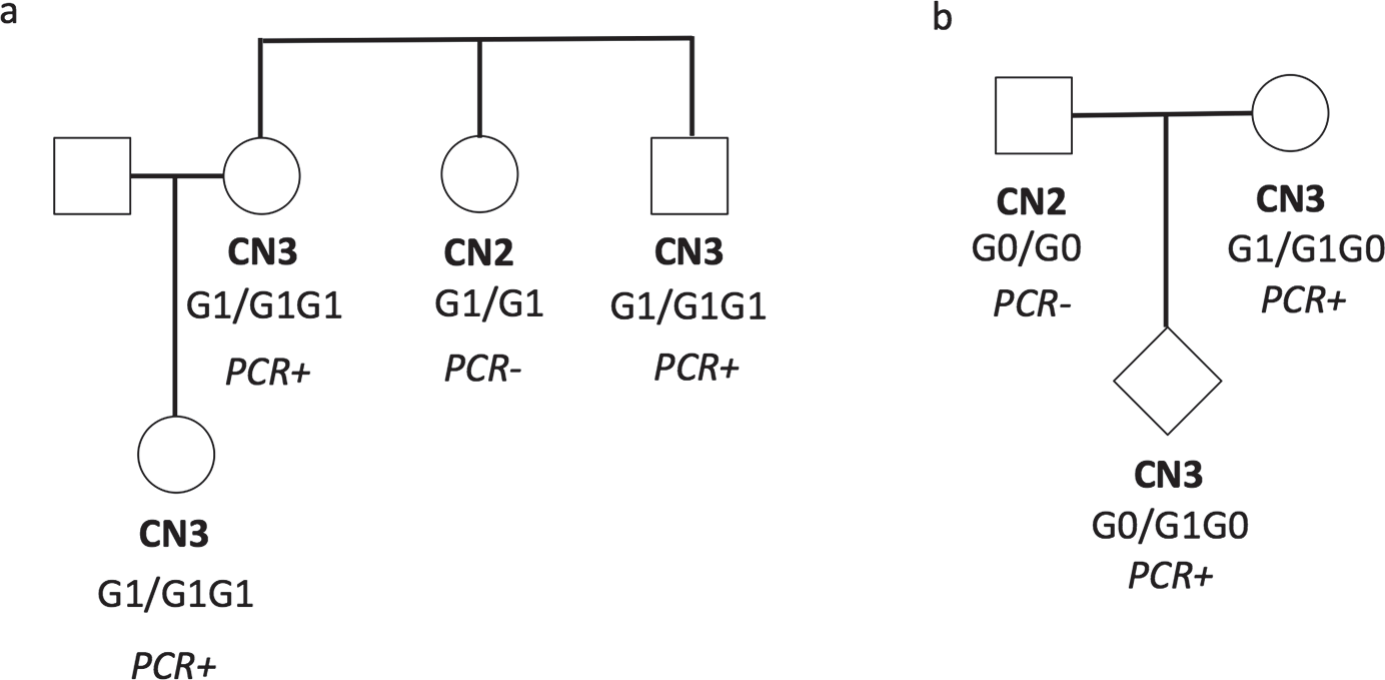
Two informative pedigrees. Pedigrees referred to in the text. CN2 or CN3 refers to 2 or 3 copies of APOL1 as determined by Taqman-based quantitative PCR assay. PCR+ or PCR- refers to presence or absence of the specific insertional event that appears to be the most common copy number variant at this locus, as detected by a PCR-based assay that amplifies the region across the insertional junction. Inferred genotype is indicated, with the two haplotypes separated by '/'.

In one branch of a large African American pedigree that we had ascertained previously because of the high rate of kidney disease in the family, we identified members of a nuclear family (woman, her brother, and her son) all with three copies of APOL1 by PCR assay. Sanger sequencing identified only G1 alleles and no G0 or G2 alleles. Therefore, these individuals all carry one haplotype with two tandem copies of the G1 form of APOL1 and one haplotype with a single APOL1 (genotype G1/G1G1, Fig 2).

We also genotyped DNA from the three members of a parent-child trio in which the mother was one of the originally identified individuals with three APOL1 copies in the 1000 Genomes dataset and an apparent G0G1 genotype (Fig 2B). Analysis of allele segregation shows that her tandem APOL1 haplotype has either G0G0 or G0G1 genotype, and her other haplotype a single copy of G1. To differentiate between these two possibilities, we used PCR to amplify a region of APOL1 containing the G1-defining SNPs, subcloned the amplified product into a plasmid vector, and sequenced several of these clones in order to determine if this individual had 2 or 1 copies of G1. Out of 24 such clones, 15 were G0 and 9 G1, suggesting that this individual was more likely to have 2 copies of G0 and 1 copy of G1, and therefore tandem duplication haplotype G0G1. A complementary assay based on relative intensities of alleles after PCR amplification and restriction digestion to determine relative amounts of the G0 and G1 alleles was consistent with this allele ratio (Fig 1D). Thus, among the samples we have examined, we have observed the tandem APOL1 duplication haplotype to have genotypes G0G0, G0G1, and G1G1.

One DNA sample (NA19372) was found to have four APOL1 copies by Taqman, and was positive for duplication by the PCR assay. PCR amplification, subcloning, and sequencing (as above) yielded 4 G1 clones and 19 G0 clones, suggesting G1G0G0G0 as the most likely genotype in this individual (Table 1).

To determine if there is concomitant alteration in transcription, we repeated the PCR fragment amplification, subcloning and sequencing using reverse transcribed RNA derived from these cell lines (S2 Table). We found that the genotype of the APOL1 transcript was consistent with that of genomic DNA, suggesting that all copies of APOL1 are being transcribed. This is a finding of significant importance since it lends credence to the possibility of alteration in functional gene dosage associated with high copy number.

## Discussion

The APOL1 gene, located on long arm of chromosome 22 (22q13.1), encodes a circulating protein, apolipoprotein-L1, with a role in both innate immunity and susceptibility to kidney disease. Two non-synonymous alleles in this gene, referred to as G1 and G2, have been shown to explain most of the high rate of non-diabetic kidney disease in people of recent African ancestry. The wild-type, or G0, form of APOL1 provides humans with protection against infection by the parental African trypanosome species, *trypanosoma brucei brucei*. The presence of one or two copies of the G1 or G2 form of APOL1 extend this effect to confer protection against *trypanosome brucei rhodesiense*, the etiologic agent of acute African sleeping sickness. The pattern of inheritance of APOL1 associated kidney disease risk is essentially autosomal recessive[3].

Copy number variation is now recognized as both a common form of human genetic variation and contributor to human phenotypic variation[14]. Chromosome 22 is especially enriched in intrachromosomal duplications, which comprise 11.9% of the whole chromosome, and is second only to Y chromosome by this measure[15,16]. Copy number variation may lead to change in dosage, and therefore expression, of a gene[17]. It has also been shown to play an important role in primate evolution[18]. The APOL1-APOL4 gene cluster on chromosome 22 is itself most likely a result of tandem duplication of an original APOL gene, believed to be what is now called APOL3, and shows a strong signature for positive selection[19, 20].

In this report, we demonstrate the presence of a 101,324 bp long duplication in long arm of chromosome 22, with coordinates 36,612,938–36,714,262 and encompassing APOL1, APOL2 and part of MYH9 genes. Initially seen on sequence coverage analysis of eight individuals with African ancestry, we confirmed the presence of this duplication by multiple experimental methods. This tandem duplication appears to be present in individuals from Africa or with recent African ancestry. As reported by Genovese et al [1], it appears that the frequency of G1 and G2 alleles rose quickly in Africa due to strong positive selection over the past several thousand years. It is likely that the APOL1 duplication event occurred during a similar time frame in Africa.

It is interesting to note the presence of a 4 bp homologous sequence at the breakpoints (TCTG at chr22:36,612,938–36,612,942 and chr22:36,714,262–36,714,266). It is too small a region of homology to account for non-allelic homologous recombination as a putative mechanism. It is therefore likely that the rearrangement occurred via a replication-based mechanism rather than a recombination based phenomenon, possibly through microhomology-mediated break-induced replication [10, 21, 22]. Sequence analysis revealed identical sequences at the junction, indicating the likelihood of a single duplication event. Irrespective of the initial APOL1 genotype of the duplicated segment, subsequent homologous recombination events over the years have probably resulted in the different combinations of G0/G1/G2 that we see today.

Analysis of the sequence of the breakpoint junction showed identical sequences in all of the samples we tested. Given the absence of the tandem duplication in people of European descent, we believe the presence of the duplication is due to a single duplication event that took place in Africa. As we believe that APOL1 alleles G1 and G2 have reached high frequency in West Africa due to natural selection, it is interesting to speculate whether the duplication event also has any beneficial effect. Since the current model predicts an increased risk for kidney disease in individuals with G1 and/or G2 in the absence of G0, pairing G0 with G1 on one chromosome would be expected to create an allele with the enhanced immune properties of the G1 in a non-risk haplotype for kidney disease.

Those individuals with the tandem duplication by the qualitative PCR assay showed 3 (or more) copies of APOL1 by a quantitative PCR based copy-number assay. However, this assay revealed extra APOL1 copies in several additional samples that were negative by the PCR assay for the common tandem duplication junction. This suggests the presence of other intragenic duplications within the APOL1 gene, consistent with the hypothesis of “duplication shadowing”, whereby loci near clusters of segmental duplication are more susceptible to further duplication and deletion events[15, 18]. Furthermore, complex genomic rearrangements are known to occur in the regions enriched in low copy repeats (LCRs) [23, 24]. As previously described by Genovese et al[3], the recombination landscape around APOL1 gene is significantly enriched in Alu elements, hence predisposing it to complex rearrangements. Our attempts to design a robust set of custom assays of copy number throughout this genomic region to better define these other CNVs was limited by the high degree of homology between genes in the APOL1-4 cluster[19, 25]. The occurrence of these small intragenic copy number variations seem to be independent of the 101,324 bp duplication event.

While the presence of two variant APOL1 alleles increases an individual’s risk of FSGS by seventeen fold, there is also a small increased risk observed in individuals with 1 risk allele. For example, individuals with one G1 allele are reported to have a 1.7 fold risk of FSGS. We wondered if structural variation in APOL1 might account for some of the variability in APOL1 G1 and G2 associated phenotypes. In particular, we were interested in whether a significant proportion of individuals genotyped as G1 or G2 heterozygotes had structural variation than might explain the slightly higher risk of kidney disease. The tandem APOL1 duplication was more common in kidney disease cases with an apparent G0G1 genotype than among controls with an apparent G0G1 genotype. When the single common tandem duplication is included in this comparison, or when we include the larger number of individuals with three or more copies of APOL1 by quantitative PCR (Taqman), this difference is statistically significant. However, additional, larger studies are required to conclude with confidence that the presence of multiple APOL1 copies contributes to kidney disease risk. Because these structural variants are rare, conducting such a study will be difficult. As a rough estimate, using the frequencies of higher copy number samples in this study and assuming controls to be twice as common as cases, a sample size of 612 (204 cases and 408 controls) will be needed to detect any difference with confidence of 99% and power of 90%[26].

We note that among individuals with extra copy of APOL1, this extra copy may be of either genotype G0 or G1. This raises the question as to whether G0G1, G1G1, and G0G0 haplotypes behave like G0 or G1, or neither. Further experiments are also needed to define whether the presence of an extra APOL1 copy alters the level of APOL1 expressed in cells and in humans.

Thus, within the complex region of the genome harboring the APOL cluster of genes, additional complexity arises from the occasional presence of additional copies of APOL1. Our evidence suggests that one such tandem duplication of approximately 100 kb of DNA containing APOL2, APOL1, and a portion of MYH9, likely occurred once within the last few thousand years in Africa. Our evidence also suggests that other duplications containing at least part of the APOL1 gene also exist at low frequency.

In the light of these findings, it will be important to perform large epidemiological studies to fully understand the exact role of such haplotypes in relation to both kidney disease and resistance to parasitic infection. Additional studies are needed to determine the functional effects of possessing more than two copies of APOL1 (and APOL2).

## Supporting Information

S1 Table Sequence data. A. Sequence data for the eight 1000 Genomes project samples discussed in the text is available online and accessible at the URLs listed. B. Sequence data (reads across DNA breakpoint) for the two relevant samples from our laboratory.
(DOCX)

S2 Table APOL1 fragment amplification and subcloning study. S2 Table, top: PCR fragment cloning and sequencing. The table shows the number of colonies that were sequenced and determined to have genotypes G0 and G1, when genomic DNA from the lymphoblast cell lines was used as template to obtain and clone the PCR fragment. The genotype was inferred in conjunction with data from Taqman copy number assay. S2 Table, bottom: Data from a similar experiment, using fragments amplified and subcloned after RT-PCR from RNA. The genotype was inferred based on the ratio of G0 to G1 colonies.
(DOCX)
