# Supplementary material for: Copy Number Variation at the APOL1 Locus
Source: PLoS One. 2015 May 1;10(5):e0125410. doi: 10.1371/journal.pone.0125410 (PMC4416782; doi:10.1371/journal.pone.0125410)
Supplement: S1 Table — A. Sequence data for the eight 1000 Genomes project samples discussed in the text is available online and accessible at the URLs listed. B. Sequence data (reads across DNA breakpoint) for the two relevant samples from our laboratory. (DOCX) [file pone.0125410.s001.docx]

**Supplementary Table 1:**

**A. Sequence data for the eight 1000 Genomes project samples discussed in the text is available online and accessible at URLs listed:**

<ftp://ftp.1000genomes.ebi.ac.uk/vol1/ftp/data/HG02284/alignment/HG02284.mapped.ILLUMINA.bwa.ACB.low_coverage.20130415.bam>

<ftp://ftp.1000genomes.ebi.ac.uk/vol1/ftp/data/HG02819/alignment/HG02819.mapped.ILLUMINA.bwa.GWD.low_coverage.20121211.bam>

<ftp://ftp.1000genomes.ebi.ac.uk/vol1/ftp/data/HG02820/alignment/HG02820.mapped.ILLUMINA.bwa.GWD.low_coverage.20121211.bam>

<ftp://ftp.1000genomes.ebi.ac.uk/vol1/ftp/data/HG03077/alignment/HG03077.mapped.ILLUMINA.bwa.MSL.low_coverage.20130415.bam>

<ftp://ftp.1000genomes.ebi.ac.uk/vol1/ftp/data/HG03518/alignment/HG03518.mapped.ILLUMINA.bwa.ESN.low_coverage.20130415.bam>

<ftp://ftp.1000genomes.ebi.ac.uk/vol1/ftp/data/NA19042/alignment/NA19042.mapped.ILLUMINA.bwa.LWK.low_coverage.20130415.bam>

<ftp://ftp.1000genomes.ebi.ac.uk/vol1/ftp/data/NA19372/alignment/NA19372.mapped.ILLUMINA.bwa.LWK.low_coverage.20120522.bam>

<ftp://ftp.1000genomes.ebi.ac.uk/vol1/ftp/data/NA19701/alignment/NA19701.mapped.ILLUMINA.bwa.ASW.low_coverage.20120522.bam>

**B. Sequence data (reads across DNA breakpoint) for the two relevant samples from our laboratory:**

| FGFM40 | @100927_HWI-ST177.PF:6:63:8771:126035/1 | TTGTTTGTTTTTAAGATACAGAATAGCTGATCTCCTATACACAAAGA |  |  |  |
| --- | --- | --- | --- | --- | --- |
| FGFM40 | @100927_HWI-ST177.PF:6:63:8771:126035/2 | CCACAACAGCCTCAACTGTGCTGCTGCAGGGACATTCACCCAGGAGG |  |  |  |
| FGFM4001 | @B00TJACXX110614:6:1104:3533:33749/1 | GTCCCCCACAGACTAAAGAGCAGGTATGTCCTCTGGGGCCAGGGCAGCTGGCTGCCTCCTGGGTGAATGTCCCTGC | | | |
| FGFM4001 | @B00TJACXX110614:6:1104:3533:33749/2 | TCTCTCCGGGTTGCTTTGATGACTTCATCATGGAATCATGTGAAAGTGCCTGACACGGACACCCCAGGATGGCCCA | | | |
| FGFM4001 | @B00TJACXX110614:6:1205:11833:45801/1 | GCCTGACACGGACACCCCAGGATGGCCCACAACAGCCTCAACTGTGCTGCTGCAGGGACATTCACCCAGGAGGCAG | | | |
| FGFM4001 | @B00TJACXX110614:6:1205:11833:45801/2 | TTCCAGGAGGTGGCAGGGAGGTGTTTTTTTGTTTGTTTTTAAGATACAGAATAGCTGATCTCCTATACACAAAGAC | | | |
| FGFM4001 | @B00TJACXX110614:6:2303:4803:63040/1 | CCCCCACAGACTAAAGAGCAGGTATGTCCTCTGGGGCCAGGGCAGCTGGCTGCCTCCTGGGTGAATGTCCCTGCAG | | | |
| FGFM4001 | @B00TJACXX110614:6:2303:4803:63040/2 | TCCGGGTTGCTGTGAGGACTGCATCATGGAATCATGTGAAAGTGCCTGACACGGACACCCCAGGATGGCCCACAAC | | | |
| FGFM4001 | @B00TJACXX110614:7:1303:20354:74062/1 | TCTATGTCCCCCACAGACTAAAGAGCAGGTATGTCCTCTGGGGCCAGGGCAGCTGGCTGCCTCCTGGGTGAATGTC | | | |
| FGFM4001 | @B00TJACXX110614:7:1303:20354:74062/2 | TGGAATCATGTGAAAGTGCCTGACACGGACACCCCAGGATGGCCCACAACAGCCTCAACTGTGCTGCTGCAGGGAC | | | |
| FGFM4001 | @B00TJACXX110614:7:2302:3930:125175/1 | TTCACCCAGGAGGCAGCCAGCTGCCCTGGCCCCAGAGGACATACCTGCTCTTTAGTCTGTGGGGGACATAGAGGGA | | | |
| FGFM4001 | @B00TJACXX110614:7:2302:3930:125175/2 | GGGAGGTGTTTTTTTGTTTGTTTTTAAGATACAGAATAGCTGATCTCCTATACACAAAGACAAAAATTCCCTCTAT | | | |
| FGFM4001 | @B00TJACXX110614:8:1105:14350:27466/1 | TGTTTTTTTGTTTGTTTTTAAGATACAGAATAGCTGATCTCCTATACACAAAGACAAAAATTCCCTCTATGTCCCC | | | |
| FGFM4001 | @B00TJACXX110614:8:1105:14350:27466/2 | TATGCCACCTCTCTCCGGGTTGCTGTGAGGACTGCATCATGGAATCATGTGAAAGTGCCTGACACGGACACCCCAG | | | |
| FGFM4001 | @B00TJACXX110614:8:1105:18659:197224/1 | CACAGACTAAAGAGCAGGTATGTCCTCTGGGGCCAGGGCAGCTGGCTGCCTCCTGGGTGAATGTCCCTGCAGCAGC | | | |
| FGFM4001 | @B00TJACXX110614:8:1105:18659:197224/2 | AAAGTGCCTGACACGGACACCCCAGGATGGCCCACAACAGCCTCAACTGTGCTGCTGCAGGGACATTCACCCAGGA | | | |
| FGFM4001 | @B00TJACXX110614:8:1202:6518:164139/1 | CCTGACACGGACACCCCAGGATGGCCCACAACAGCCTCAACTGTGCTGCTGCAGGGACATTCACCCAGGAGGCAGC | | | |
| FGFM4001 | @B00TJACXX110614:8:1202:6518:164139/2 | GTTTTTTTGTTTGTTTTTAAGATACAGAATAGCTGATCTCCTATACACAAAGACAAAAATTCCCTCTATGTCCCCC | | | |
| FGFM4001 | @B00TJACXX110614:8:2301:6592:167603/1 | CCACAGACTAAAGAGCAGGTATGTCCTCTGGGGCCAGGGCAGCTGGCGGCCTCCTGGGTGAATGTCCCTGCAGCAG | | | |
| FGFM4001 | @B00TJACXX110614:8:2301:6592:167603/2 | CTTGATGACCAATCAGCCAAAATACCGTCTCAATGTAAAGAGGCACAATGCAGTCTCAGGATGGCACTTCCTGGGC | | | |
| FGFM4001 | @B086BABXX110425:2:1203:19598:100933/1 | TCCCCCACAGACTAAAGAGCAGGTATGTCCTCTGGGGCCAGGGCAGCTGGCTGCCTCCTGGGTGAATGTCCCTGCA | | | |
| FGFM4001 | @B086BABXX110425:2:1203:19598:100933/2 | CTCCAGTGCTCTAGATGGGGTTATGCCACCTCTCTCCGGGTTGCTGTGAGGACTGCATCATGGAATCATGTGAAAG | | | |
| FGFM4001 | @B086BABXX110425:3:1206:1832:139358/1 | CCCCCACAGACTAAAGAGCAGGTATGTCCTCTGGGGCCAGGGCAGCTGGCTGCCTCCTGGGTGAATGTCCCTGCAG | | | |
| FGFM4001 | @B086BABXX110425:3:1206:1832:139358/2 | AATCATGTGAAAGTGCCTGACACGGACACCCCAGGATGGCCCACAACAGCCTCAACTGTGCTGCTGCAGGGACATT | | | |
| FGFM4001 | @B086BABXX110425:4:1101:11856:104891/1 | GCATCATGGAATCATGTGAAAGTGCCTGACACGGACACCCCAGGATGGCCCACAACAGCCTCAACTGTGCTGCTGC | | | |
| FGFM4001 | @B086BABXX110425:4:1101:11856:104891/2 | AAAATTCCCTCTATGTCCCCCACAGACTAAAGAGCAGGTATGTCCTCTGGGGCCAGGGCAGCTGGCTGCCTCCTGG | | | |
| FGFM4001 | @B086BABXX110425:4:2105:7604:109252/1 | CCCCAGGATGGCCCACAACAGCCTCAACTGTGCTGCTGCAGGGACATTCACCCAGGAGGCAGCCAGCTGCCCTGGC | | | |
| FGFM4001 | @B086BABXX110425:4:2105:7604:109252/2 | TACAGAATAGCTGATCTCCTATACACAAAGACAAAAATTCCCTCTATGTCCCCCACAGACTAAAGAGCAGGTATGT | | | |
| FGFM4001 | @B086BABXX110425:4:2202:3334:117962/1 | AGTGCCTGACACGGACACCCCAGGATGGCCCACAACAGCCTCAACTGTGCTGCTGCAGGGACATTCACCCAGGAGG | | | |
| FGFM4001 | @B086BABXX110425:4:2202:3334:117962/2 | AGACAAAAATTCCCTCTATGTCCCCCACAGACTAAAGAGCAGGTATGTCCTCTGGGGCCAGGGCAGCTGGCTGCCT | | | |
| FGFM4001 | @B086BABXX110425:5:2104:6899:53714/1 | TTGCTGTGAGGACTGCATCATGGAATCATGTGAAAGTGCCTGACACGGACACCCCAGGATGGCCCACAACAGCCTC | | | |
| FGFM4001 | @B086BABXX110425:5:2104:6899:53714/2 | AGGAGGTGGCAGGGAGGTGTTTTTTTGTTTGTTTTTAAGATACAGAATAGCTGATCTCCTATACACAAAGACAAAA | | | |
| FGFM4001 | @B09N0ABXX110520:1:1105:5455:190209/1 | CCCCCACAGACTAAAGAGCAGGTATGTCCTCTGGGGCCAGGGCAGCTGGCTGCCTCCTGGGTGAATGTCCCTGCAG | | | |
| FGFM4001 | @B09N0ABXX110520:1:1105:5455:190209/2 | GGACACCCCAGGATGGCCCACAACAGCCTCAACTGTGCTGCTGCAGGGACATTCACCCAGGAGGCAGCCAGCTGCC | | | |
| FGFM4001 | @B09N0ABXX110520:1:1107:3212:82840/1 | TCCGGGTTGCTGTGAGGACTGCATCATGGAATCATGTGAAAGTGCCTGACACGGACACCCCCGGATGGCCCCCAAC | | | |
| FGFM4001 | @B09N0ABXX110520:1:1107:3212:82840/2 | GACTTAACCATGGGCGCCCGAGGGGTCTTCCAGGAGGTGGCAGGGAGGTGTTTTTTTGTTTGTTTTTAAGATACAG | | | |
| FGFM4001 | @B09N0ABXX110520:1:2205:15693:31652/1 | ACAAAAATTCCCTCTATGTCCCCCACAGACTAAAGAGCAGGTATGTCCTCTGGGGCCAGGGCAGCTGGCTGGCTCC | | | |
| FGFM4001 | @B09N0ABXX110520:1:2205:15693:31652/2 | AGGATGGCCCACAACAGCCTCAACTGTGCTGCTGCAGGGACATTCACCCAGGAGGCAGCCAGCTGCCCTGGCCCCA | | | |
| FGFM4001 | @B09N0ABXX110520:2:2108:1851:129463/1 | CCACAGACTAAAGAGCAGGTATGTCCTCTGGGGCCAGGGCAGCTGGCTGCCTCCTGGGGGAATGTCCCTGCAGCAG | | | |
| FGFM4001 | @B09N0ABXX110520:2:2108:1851:129463/2 | CTGACACGGACACCCCAGGATGGCCCACAACAGCCTCAACTGTGCTGCTGCAGGGACATTCACCCAGGAGGCAGCC | | | |
| FGFM4001 | @B09N0ABXX110520:3:2108:5112:47063/1 | TTCTCCAGTGCTCTAGATGGGGTTATGCCACCTCTCTCCGGGTTGCTGTGAGGACTGCATCATGGAATCATGTGAA | | | |
| FGFM4001 | @B09N0ABXX110520:3:2108:5112:47063/2 | TATGTCCCCCACAGACTAAAGAGCAGGTATGTCCTCTGGGGCCAGGGCAGCTGGCTGCCTCCTGGGTGAATGCCGC | | | |
| FGFM4001 | @B0BNFABXX110425:1:2202:11724:165893/1 | GACATTCACCCAGGAGGCAGCCAGCTGCCCTGGCCCCAGAGGACATACCTGCTCTTTAGTCTGTGGGGGACATAGA | | | |
| FGFM4001 | @B0BNFABXX110425:1:2202:11724:165893/2 | CAGGACATACAGGTGATATTTCTGGTGTTTGAATTCTACCAAAGGCAACCTTCCTAGTGAAACCAATAAGCCTGAA | | | |
| FGFM4001 | @B0BNFABXX110425:2:2108:6414:181197/1 | CCGAGGGGTCTTCCAGGAGGTGGCAGGGAGGTGTTTTTTTGTTTGTTTTTAAGATACAGAATAGCTGATCTCCTAT | | | |
| FGFM4001 | @B0BNFABXX110425:2:2108:6414:181197/2 | CGGACACCCCAGGATGGCCCACAACAGCCTCAACTGTGCTGCTGCAGGGACATTCACCCAGGAGGCAGCCAGCTGC | | | |
| FGFM4001 | @B0BNFABXX110425:4:1108:10873:191411/1 | TCCAGGAGGTGGCAGGGAGGTGTTTTTTTGTTTGTTTTTAAGATACAGAATAGCTGATCTCCTATACACAAAGACA | | | |
| FGFM4001 | @B0BNFABXX110425:4:1108:10873:191411/2 | GGATGGCCCACAACAGCCTCAACTGTGCTGCTGCAGGGACATTCACCCAGGAGGCAGCCAGCTGCCCTGGCCCCAG | | | |
| FGFM4001 | @B0BNFABXX110425:8:2205:19277:172489/1 | TTATGCCACCTCTCTCCGGGTTGCTGTGAGGACTGCATCATGGAATCATGTGAAAGTGCCTGACACGGACACCCCA | | | |
| FGFM4001 | @B0BNFABXX110425:8:2205:19277:172489/2 | TCCCCCACAGACTAAAGAGCAGGTATGTCCTCTGGGGCCAGGGCAGCTGGCTGCCTCCTGGGTGAATGTCCCTGCA | | | |
| FGFM4001 | @C02VYABXX110425:2:1205:1876:74642/1 | CCCCACAGACTAAAGAGCAGGTATGTCCTCTGGGGCCAGGGCAGCTGGCTGCCTCCTGGGTGAATGTCCCTGCAGC | | | |
| FGFM4001 | @C02VYABXX110425:2:1205:1876:74642/2 | TGCCATGAAGCTTCTCCAGTGCTCTAGATGGGGTTATGCCACCTCTCTCCGGGTTGCTGTGAGGACTGCATCATGG | | | |
| FGFM4001 | @C02VYABXX110425:2:2105:6617:194207/1 | AAAAATTCCCTCTATGTCCCCCACAGACTAAAGAGCAGGTATGTCCTCTGGGGCCAGGGCAGCTGGCTGCCTCCTG | | | |
| FGFM4001 | @C02VYABXX110425:2:2105:6617:194207/2 | CCATGAAGCTTCTCCAGTGCTCTAGATGGGGTTATGCCACCTCTCTCCGGGTTGCTGTGAGGACTGCATCATGGAA | | | |
| FGFM4001 | @C02VYABXX110425:2:2106:8583:61637/1 | AGGGAGGTGTTTTTTTGTTTGTTTTTAAGATACAGAATAGCTGATCTCCTATACACAAAGACAAAAATTCCCTCTA | | | |
| FGFM4001 | @C02VYABXX110425:2:2106:8583:61637/2 | CCCAGGATGGCCCACAACAGCCTCAACTGTGCTGCTGCAGGGACATTCACCCAGGAGGCAGCCAGCTGCCCTGGCC | | | |
| FGFM4001 | @C02VYABXX110425:3:1207:6348:187709/1 | ACAGAATAGCTGATCTCCTATACACAAAGACAAAAATTCCCTCTATGTCCCCCACAGACTAAAGAGCAGGTATGTC | | | |
| FGFM4001 | @C02VYABXX110425:3:1207:6348:187709/2 | CTGCAGGGACATTCACCCAGGAGGCAGCCAGCTGCCCTGGCCCCAGAGGACATACCTGCTCTTTAGTCTGTGGGGG | | | |
| FGFM4001 | @C02VYABXX110425:4:1204:12823:109100/1 | CTATGTCCCCCACAGACTAAAGAGCAGGTATGTCCTCTGGGGCCAGGGCAGCTGGCTGCCTCCTGGGTGAATGTCC | | | |
| FGFM4001 | @C02VYABXX110425:4:1204:12823:109100/2 | CATCATGGAATCATGTGAAAGTGCCTGACACGGACACCCCAGGATGGCCCACAACAGCCTCAACTGTGCTGCTGCA | | | |
| FGFM4001 | @C02VYABXX110425:7:1102:5045:159458/1 | GGCCCACAACAGCCTCAACTGTGCTGCTGCAGGGACATTCACCCAGGAGGCAGCCAGCTGCCCTGGCCCCAGAGGA | | | |
| FGFM4001 | @C02VYABXX110425:7:1102:5045:159458/2 | CCCTCTATGTCCCCCACAGACTAAAGAGCAGGTATGTCCTCTGGGGCCAGGGCAGCTGGCTGCCTCCTGGGTGAAT | | | |
| FGFM4001 | @C02VYABXX110425:7:1205:11753:108725/1 | AGTAAGGTTGTGACTTAACCATGGGCGCCCGAGGGGTCTTCCAGGAGGTGGCAGGGAGGTGTTTTTTTGTTTGTTT | | | |
| FGFM4001 | @C02VYABXX110425:7:1205:11753:108725/2 | GACTGCATCATGGAATCATGTGAAAGTGCCTGACACGGACACCCCAGGATGGCCCACAACAGCCTCAACTGTGCTG | | | |
| FGFM4001 | @C02VYABXX110425:7:1207:17210:94131/1 | TGTGCTGCTGCAGGGACATTCACCCAGGAGGCAGCCAGCTGCCCTGGCCCCAGAGGACATACCTGCTCTTTAGTCT | | | |
| FGFM4001 | @C02VYABXX110425:7:1207:17210:94131/2 | AAGTAAGGTTGTGACTTAACCATGGGCGCCCGAGGGGTCTTCCAGGAGGTGGCAGGGAGGGGTTTTTTTGTTTGTT | | | |
| FGFM4001 | @C02VYABXX110425:7:2108:3965:147040/1 | GACACGGACACCCCAGGATGGCCCACAACAGCCTCAACTGTGCTGCTGCAGGGACATTCACCCAGGAGGCAGCCAG | | | |
| FGFM4001 | @C02VYABXX110425:7:2108:3965:147040/2 | ACAAAAATTCCCTCTATGTCCCCCACAGACTAAAGAGCAGGTATGTCCTCTGGGGCCAGGGCAGCTGGCTGCCTCC | | | |
