# Supplementary material for: Copy Number Variation at the APOL1 Locus
Source: PLoS One. 2015 May 1;10(5):e0125410. doi: 10.1371/journal.pone.0125410 (PMC4416782; doi:10.1371/journal.pone.0125410)
Supplement: S2 Table — , top: PCR fragment cloning and sequencing. The table shows the number of colonies that were sequenced and determined to have genotypes G0 and G1, when genomic DNA from the lymphoblast cell lines was used as template to obtain and clone the PCR fragment. The genotype was inferred in conjunction with data from Taqman copy number assay. S2 Table, bottom: Data from a similar experiment, using fragments amplified and subcloned after RT-PCR from RNA. The genotype was inferred based on the ratio of G0 to G1 colonies. (DOCX) [file pone.0125410.s002.docx]

**Supplementary Table 2**

| Cell Line | Number of G0 colonies | Number of G1 colonies | Total number of colonies | Inferred genotype |
| --- | --- | --- | --- | --- |
| NA 19701 | 22 | 14 | 36 | G0G0G1 |
| NA 19702 | 19 | 17 | 36 | G0G1 |
| NA 19372 | 26 | 10 | 36 | G0G0G0G1 |

**APOL1 fragment amplification and subcloning study**

| Cell Line | Number of G0 colonies | Number of G1 colonies | Total number of colonies | Copy number by Taqman | Inferred genotype |
| --- | --- | --- | --- | --- | --- |
| NA 19701 | 15 | 9 | 24 | 3 | G0G0G1 |
| NA 19702 | 12 | 11 | 23 | 2 | G0G1 |
| NA 19372 | 19 | 4 | 23 | 4 | G0G0G0G1 |

*APOL1 genomic DNA amplification and fragment subcloning*

*APOL1 RT-PCR from RNA and fragment subcloning*

Supplementary Table 2, top: PCR fragment cloning and sequencing. The table shows the number of colonies that were sequenced and determined to have genotypess G0 and G1, when genomic DNA from the lymphoblast cell lines was used as template to obtain and clone the PCR fragment. The genotype was inferred in conjunction with data from Taqman copy number assay.

Supplementary Table 2, bottom: Data from a similar experiment, using fragments amplified and subcloned after RT-PCR from RNA. The genotype was inferred based on the ratio of G0 to G1 colonies.
